# The Effects of a High-Protein Dairy Milk Beverage With or Without Progressive Resistance Training on Fat-Free Mass, Skeletal Muscle Strength and Power, and Functional Performance in Healthy Active Older Adults: A 12-Week Randomized Controlled Trial

**DOI:** 10.3389/fnut.2021.644865

**Published:** 2021-03-17

**Authors:** Zoya Huschtscha, Alexandra Parr, Judi Porter, Ricardo J. S. Costa

**Affiliations:** ^1^Department of Nutrition Dietetics & Food, Monash University, Notting Hill, VIC, Australia; ^2^School of Exercise and Nutrition Sciences, Institute for Physical Activity and Nutrition (IPAN), Deakin University, Geelong, VIC, Australia

**Keywords:** leucine, calcium, inflammatory cytokines, insulin, insulin-like growth factor, testosterone, estradiol, cortisol

## Abstract

The study aimed to investigate the independent and combined effects of consuming a high-protein dairy milk beverage, twice daily, with or without a progressive resistance training (PRT) program on outcomes of age-related sarcopenia, in healthy active older (≥50 years) adults. In this 12-week, 2 × 2 factorial study, participants were randomly allocated into one of four groups: dairy milk beverage (DM), exercise and dairy milk beverage (EX+DM), exercise alone (EX), and control (CON). The EX group underwent a 12-week whole-body PRT schedule (three sessions/week) and a high-protein dairy milk beverage (DM) was consumed twice daily (30 g protein/day). At weeks 0, 6, and 12, body composition (iDXA), strength [one-repetition maximum (1RM): leg press, chest press, lateral (*lat*) pull-down, and handgrip], power (countermovement jump), cardiorespiratory fitness (*V*O_2_), and physical performance (gait speed) were measured. Before measurements, blood samples were collected to determine the immune (i.e., leukocyte trafficking and inflammatory cytokines) and hormonal (i.e., insulin, cortisol, IGF-1, testosterone, and estradiol) profiles. Participants (*n* = 37) completed the study within the controlled experimental conditions. Protein intake increased in the EX+DM [mean ± SD, 1.2 ± 0.2 to 1.8 ± 0.4 g/kg body mass (BM) per day^−1^] and DM (1.3 ± 0.5 to 1.8 ± 0.6 g kg^−1^ BM day^−1^) groups during the intervention. Absolute fat-free mass increased in the EX+DM [mean (95% confidence interval) = 0.65 (0.25–1.0) kg] and EX [0.49 (−0.44 to 1.40) kg] groups (*P* < 0.001) compared to DM [−0.54 (−1.6 to 0.05) kg]. Relative fat mass decreased (group^*^time, *P* = 0.018) in DM [−1.8% (−3.3 to −0.35%)] and EX+DM [−1.3% (−2.3 to −0.31%)], which was a greater reduction than that in the CON [0.10% (−0.80 to 1.0%)] group (P < 0.01). Relative maximal strength increased in both the EX and EX+DM (≥35%, *P* < 0.05) groups, but not in the DM and CON groups. The change in 1RM strength outcomes was higher in EX+DM compared to all other groups (53–78%, *P* < 0.01). There was an increase in resting plasma IL-10 concentration in EX+DM (88%), compared to all the other groups (*P* = 0.016). No other differences in systemic inflammatory cytokines were observed. There were no significant changes in all hormone concentrations measured among all groups. In conclusion, a high-protein dairy milk beverage providing additional protein did not further enhance the effects of PRT on outcomes of fat-free mass, power, or physical performance. However, there was a significant augmentative effect for high-protein dairy milk consumption on changes to maximal strength outcomes during PRT in healthy active older adults.

## Introduction

There has been considerable research exploring the age-related decline in skeletal muscle mass and function (e.g., strength, power, and performance measures), collectively known as sarcopenia (1, 2). The multifactorial (e.g., training status, biological sex, age, and nutrition status) and dynamic (e.g., hormonal and immunological) pathophysiological process of sarcopenia is complex, and currently, there is limited evidence to support the efficacy of pharmacological treatments (3, 4). Therefore, there has been an increased interest in modifiable lifestyle factors such as exercise (e.g., resistance training) and nutrition (e.g., dietary protein) for the treatment and management of age-related sarcopenia. To date, there have been numerous randomized controlled trials and meta-analyses that have consistently reported that progressive resistance training (PRT) can effectively improve gains in fat-free mass (FFM) (~1.2 kg), maximal strength (≥25%), and physical functional performance (e.g., gait speed) in older adults (>50 years) (5–7). There is no consensus regarding the effect of protein supplementation [e.g., whey protein, casein, essential amino acids (EAAs), and/or leucine] on augmenting further adaptations of FFM, skeletal muscle strength, and power following PRT (8–10). Many studies are confounded by the inclusion of frail institutionalized or sedentary community-dwelling adults, often referred to as “older adults,” who are predominantly aged ≥60 years, the varied use of supplementation (e.g., type, form, dose, and frequency) and outcome measures, and the large variations of baseline habitual protein intakes (2, 8, 9). Active older adults (≥50 years) who regularly engage in physical activity—from 150 min/week of light-intensity [e.g., 3–5 metabolic equivalents (METs)] to moderate-intensity (e.g., 6–9 METs) physical activity or 75 min/week of vigorous-intensity (e.g., >9 METS) physical activity (11), either recreationally or competitively—still show signs of age-related sarcopenia (12). Although active older adults do not have the confounding variables attributed to frailty, sedentary behavior, and/or disease (pathogenic hormonal and/or inflammatory status), they are currently underrepresented in sarcopenia research. While ≥50 years is not considered “older” in the spectrum of sarcopenia research, it is the age at which sarcopenia begins to be noticeable (5–7). Furthermore, given the potential efficacy of pairing PRT with protein supplementation, nutritional interventions in active older adults are limited and require further exploration in order to examine their effectiveness in this population.

Higher daily protein intakes [>1.2 g/kg body mass (BM) per day] have been suggested for active older adults, exceeding the current recommended dietary allowance (RDA) of protein (e.g., 0.8 g kg^−1^ BM day^−1^), to overcome the increased requirements of amino acid utilization from the exercise stimulus and the blunted response to muscle protein synthesis (MPS) known as “anabolic resistance” (13, 14). Moreover, evenly distributed relative and absolute protein intakes per meal have been advocated, as there is an observed maximum capacity for the utilization of EAAs (15). Optimal doses of protein per meal to elicit a near-maximal response have been reported at ~25–35 g/meal (~10 g EAA) (16, 17) or relative amounts of 0.40 g/kg BM per meal (18). Numerous cross-sectional studies have observed skewed distributions of protein intake across the day, often not reaching the adequate threshold at breakfast and lunchtime for older adults (19, 20). The unevenness of the protein distribution across the day has been associated with higher levels of frailty (21) in older (≥75 years) community-dwelling individuals. However, in a cohort of “healthier” older adults (75–85 years), there were no observed associations between protein distribution and the outcomes of skeletal muscle mass and strength (22). Noting that these findings are mostly drawn from observational research, there is a lack of data from randomized controlled trials supporting the consumption of ≥1.2 g kg^−1^ BM day^−1^ in a dietary intake with a balanced protein distribution on outcomes of skeletal muscle mass and physical function in healthy active older adults.

The majority of recent research regarding protein requirements for older adults derives from single-type protein supplementation sources [e.g., whey protein isolate; (9, 10)]. However, there has been increased interest in the use of whole foods (e.g., dairy milk) as a protein source to facilitate gains in skeletal muscle mass and strength with PRT in older adults (23). Dairy milk (e.g., bovine), which comprises both whey (20%) and casein (80%), is considered a high-quality protein source as it contains all the EAAs and high levels of leucine (24). There is limited research using dairy milk alone, without additional fortification, in older adults (25–28). Studies in younger athletic populations have shown promising outcomes using unfortified dairy milk beverages on outcomes of skeletal muscle mass, strength, and physical function (29, 30). Despite these observed benefits of dairy milk beverages in younger active adults, an investigation of the effects of dairy milk on these same outcomes in active older adults remains a research gap.

Aging is characterized by a decline in anabolic hormones [e.g., testosterone and insulin-like growth factor-1 (IGF-1)] and a state of chronic low-grade inflammation, a term known as “inflammaging” (31, 32). Raised levels of systemic inflammatory cytokines, such as tumor necrosis factor-α (TNF-α) and interleukin (IL)-6, and a reduction of anti-inflammatory cytokines such as IL-10 are a common feature (31, 32). Low-grade inflammation in older adults has been associated with the acceleration of the aging process, leading to a decline in skeletal muscle mass and function (32, 33). In multiple observational studies, older adults (>60 years) have an inverse dose–response relationship between physical activity and systemic inflammatory biomarkers even at modest activity levels (33), whereas in exercise intervention studies that have provided resistance training, raised plasma concentrations of IL-10 have been reported following 16–24 weeks of training in older adults (34, 35), suggesting that exercise has the ability to prompt anti-inflammatory processes. Considering that dairy milk contains components associated with anti-inflammatory (e.g., casein-derived bioactive peptides) and immunomodulatory effects, together with resistance training, this combination may act synergistically to reduce inflammaging (23). However, the majority of studies exploring this interaction are mainly based on observational findings with limited evidence on the effects of dietary and exercise interventions on anabolic and cytokine outcomes.

The current study aimed to determine the independent and combined effects of a high-protein dairy milk beverage provided at breakfast and lunch (or after resistance exercise), with or without PRT, on outcomes of FFM, skeletal muscle strength and power, and physical performance in a cohort of healthy active older adults. We sought to evaluate the study hypothesis that providing a high-protein milk on its own would maintain outcomes of FFM, skeletal muscle strength and power, and physical performance compared to those that receive no intervention (e.g., control). In comparison, we hypothesized that a high-protein dairy milk beverage in conjunction with PRT would further enhance the effects of PRT, leading to augmented gains in FFM and skeletal muscle strength and power and an improved physical performance compared to PRT alone.

## Materials and Methods

The study protocol obtained approval from the Monash University Human Research Ethics Committee (project number 12812), in accordance with the Helsinki Declaration for human research ethics. Informed written consent was obtained from all participants before they were enrolled in the trial. The study was registered with the Australian and New Zealand Clinical Trial Registry as ANZCT12618001088235.

### Participants and Study Design

Older adult males and females (≥50 years old, with no age upper limit) performing exercise training for recreational fitness and/or sports competitions (e.g., endurance runners or aerobic gym goers) three or more structured exercise sessions per week, totaling ≥90 min/week of structured exercise duration, plus additional unstructured physical activity that accounted for meeting the Australian physical activity guidelines (36), were recruited from metropolitan Melbourne and surrounding areas in Victoria, Australia. Interested participants were initially screened over the telephone and excluded based on the following criteria: (1) dairy protein allergy or known lactose intolerances; (2) currently using dietary protein supplements; (3) any injuries preventing safe exercise; (4) had surgery in the past 12 months; (5) had an acute coronary (e.g., myocardial infarction) or vascular event in the last year, as well as uncontrolled coronary heart disease; (6) had a stroke in the past 2 years; (7) have orthopedic limitations that limit participation in the exercise program; (8) been diagnosed with or taking medication for thyroid condition; (9) had weight loss of more than 5% of body weight over the last 6 months; (10) take medications that could interfere with skeletal muscle mass structure and/or function (e.g., corticosteroids, testosterone replacement, or anabolic drugs); (11) currently undergoing immunosuppressive therapy or hormone replacement therapy; (12) have any chronic diseases, such as diabetes mellitus or gastrointestinal diseases/disorders; (13) consume more than two standard drinks of alcohol/day or 14 drinks of alcohol/week; (14) were a smoker; (15) had a BMI >30 kg/m^2^; and (16) had participated in a structured resistance training program in the past 12 months. Once participants were deemed eligible, data were collected during the period from September 2018 to January 2020.

**Figure 1:**
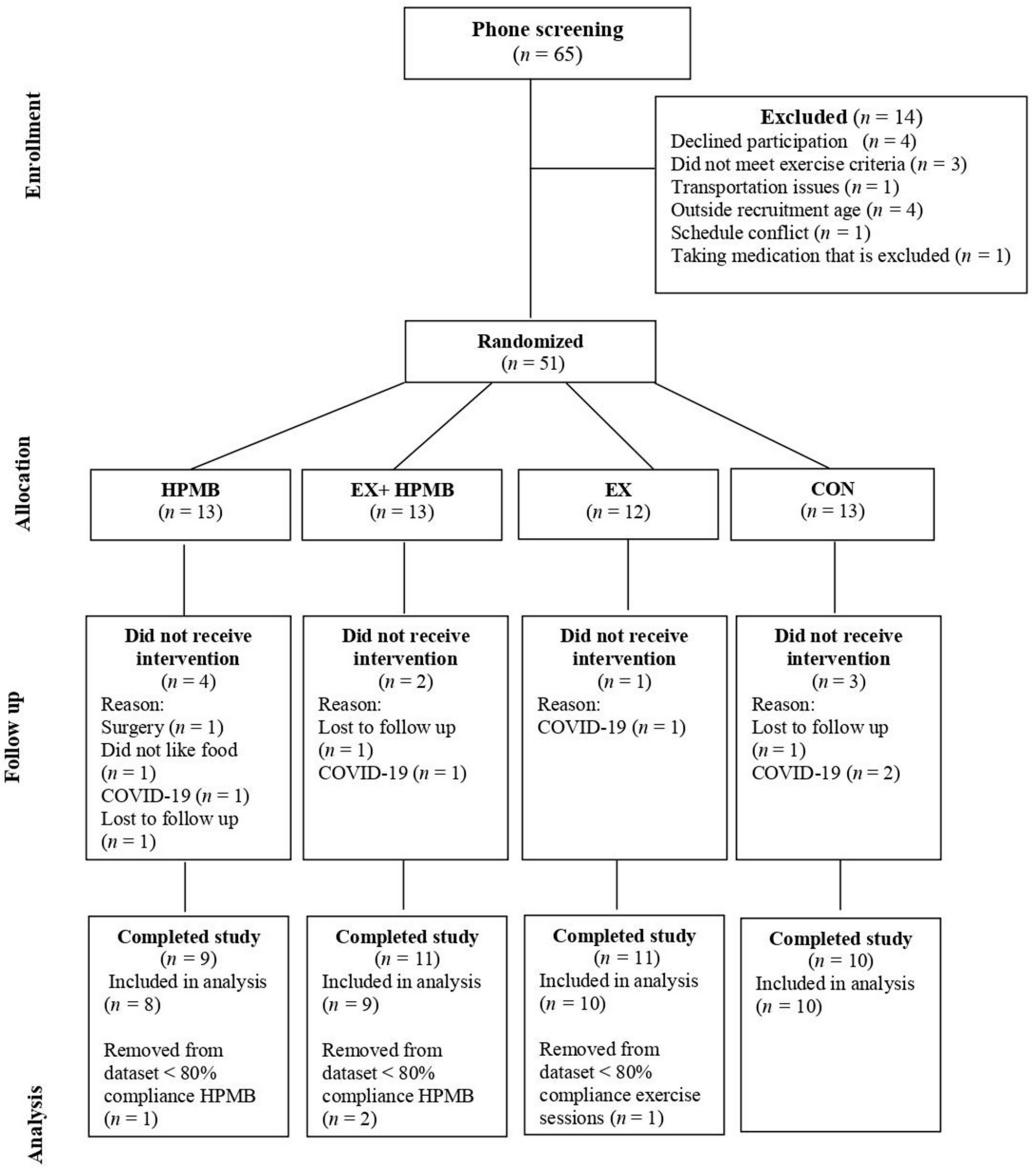
Flow diagram for participant identification, screening, eligibility, and completion.

A total of 65 participants expressed interest in participating; of these, 51 were eligible to participate and were randomly assigned into one of four groups: high-protein dairy milk beverage alone (DM), exercise and high-protein dairy milk beverage (EX+DM), exercise alone (EX), and control (CON) (Figure 1). Participants in CON were free-living, with self-selected physical activity and food/fluid intakes that were assessed in the laboratory at baseline and at 6 and 12 weeks as per the other groups. Randomization was carried out by a researcher blinded to the allocation using a block randomization table scheme with stratification by age and sex. Of the 51 randomized participants, five ceased the trial due to restrictions imposed by the COVID-19 pandemic. Other reasons for withdrawal from the study are provided in Figure 1. Due to the timeline of the data and sample collection, participants did not liaise with each other within or outside the experimental procedures. In case of close contact with participants (e.g., crossover time during PRT), the participants were advised not to discuss study participation with others.

### Preliminary Data

Prior to commencing any physical activity, participants filled out a physical activity readiness questionnaire (PAR-Q), in which they self-reported their level of activity including exercise volume and type. The participants were asked to complete a 3-day food–fluid diary prior to their baseline visit, and again at 6 and 12 weeks during the intervention, as previously described (37). Food–fluid diaries were analyzed using FoodWorks v.10.0 nutritional analysis software (Xyris Software, 2019, Brisbane, Australia) based on the Australian Food Composition Database (AFCD) 2019. Total energy, macronutrients, and calcium intake were obtained, and then dietary protein intakes distributed across breakfast, lunch, dinner, and snacks were extracted. Protein intakes per meal and per day were expressed as absolute (i.e., grams per day or grams per meal) and relative to body mass (i.e., grams per kilogram BM per day and grams per kilogram BM per meal).

### High-Protein Dairy Milk Beverage

**Figure 2:**
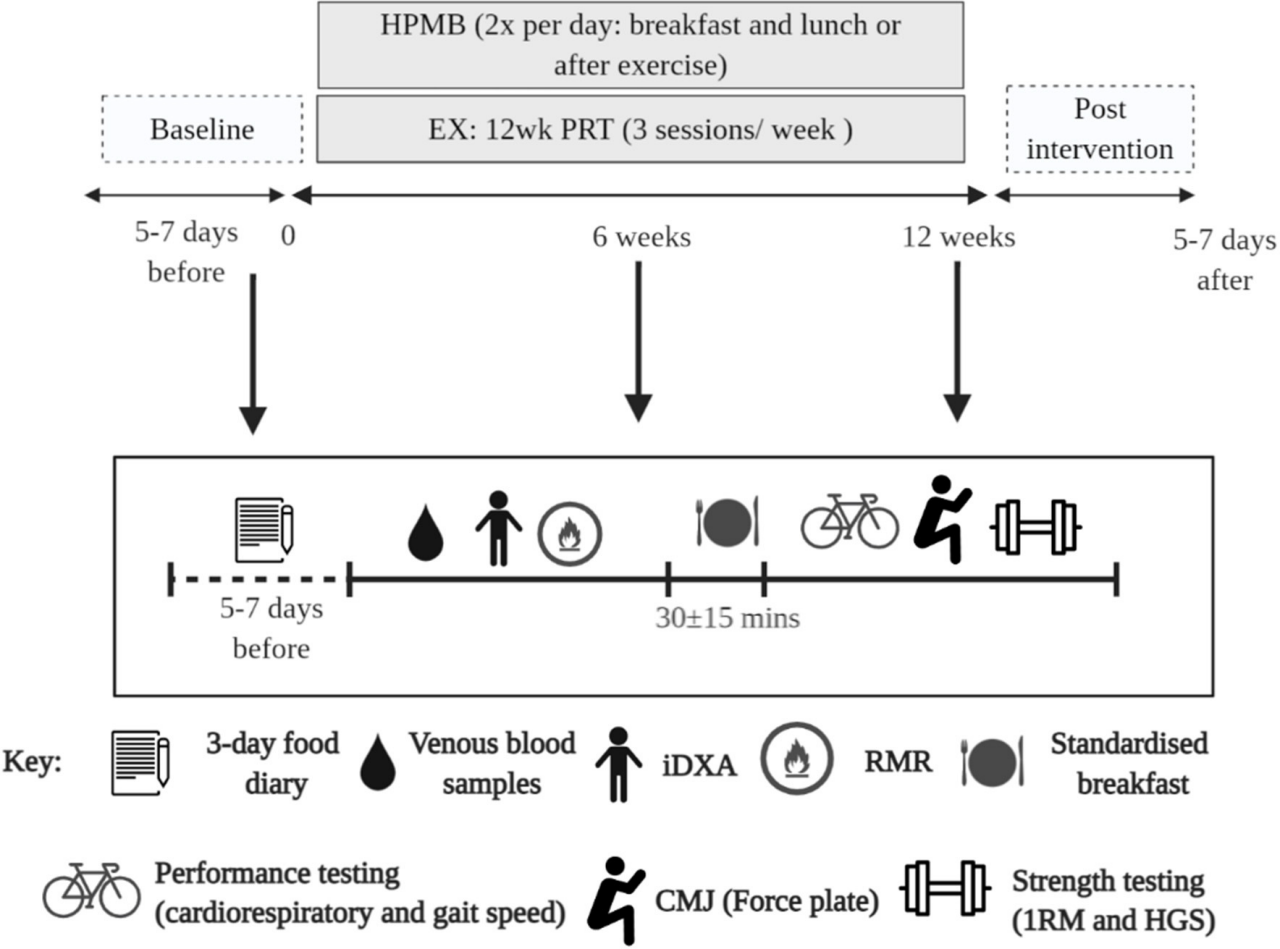
Schematic illustration of the experimental procedures and study. DM, high-protein milk beverage; EX+DM, exercise + high-protein milk beverage; EX, exercise; CON, control.

Participants assigned to DM and EX+DM were asked to consume 500 ml/day (2 × 250 ml) of reduced fat (1.5%) fresh dairy milk (Complete Dairy, Lion Dairy & Drinks, Melbourne, Australia). Participants were provided with a measuring cup and asked to consume 250 ml of dairy milk in the morning (with breakfast) and another at lunchtime (or supervised after resistance exercise in EX+DM, consumed within 10 min of completing their session). Each 250-ml cup of dairy milk contained: 535 kJ energy, 15.0 g protein (1.57 g leucine), 8.3 g carbohydrates (8.3 g lactose), 3.8 g fat, and 435 mg calcium. The participants received food provisions to deliver 100% of the total daily estimated energy requirements and 100% of the total daily estimated protein requirements (~1.2 g kg^−1^ BM day^−1^) over the entire duration of the experimental procedure, facilitated by an accredited practicing dietitian. Energy requirements were calculated using the participants' resting metabolic rate (RMR) scaled by an activity factor based on reported exercise (1.37 ± 0.09). RMR was determined by indirect calorimeter (Vmax Encore Metabolic Cart, Carefusion, San Diego, CA) in temperate ambient conditions (22.2 ± 1.4°C) and in accordance with best practice guidelines (38). Intake compliance of daily food provisions and dairy milk, and consumption of other foods/fluids, was recorded using a food–fluid diary. Milk bottles were returned weekly prior to collecting the participants' subsequent week's dairy milk and food provisions. An outline of the study protocol is depicted in Figure 2.

### Exercise Protocol

Participants allocated to EX+DM and EX were required to attend supervised PRT sessions, on three non-consecutive days per week, for 12 weeks at the research laboratory. These sessions were conducted either in a morning session (7:00 a.m. to 10:00 a.m.) or afternoon session (1:00 p.m. to 3:00 p.m.), to account for participants' work–life schedule. During the course of the trial, all exercise sessions were instructed by a strength and conditioning qualified investigator to ensure correct lifting, to monitor the appropriate amount of exercise and rest intervals, and to check compliance. Each training session (30 ± 15 min) consisted of full-body resistance training, which included leg press, *latissimus dorsi* (*lat*) pull-down, and chest press (Hammer Strength, LifeFitness, Sydney, Australia). Additional exercises including biceps curls, triceps extensions, shoulder raises, calf raises, cable deadlifts, leg curls, back rows, and abdominal exercises were used on a cable machine (Infinity Series Functional Trainer, Keiser, Fresno, CA) and rotated throughout the program to ensure the development of muscle balance. During the first 2 weeks of the PRT program, the participants completed three sets of 10–15 repetitions of 50–60% of their one-repetition maximum (1RM) with 2-min rest intervals. For the following 4 weeks, the training volume was set at three sets of 8–12 repetitions at an intensity of 69–75% of 1RM, which increased to 80–95% for six to eight repetitions for the remaining 6 weeks. Each week, weight progressively increased by 5–10%. For all exercises, the participants were instructed to perform each repetition in a slow, controlled manner, with a rest of 2 min between sets. Testing for participants' 1RM occurred at baseline and week 6; weights were adjusted according to their new 1RM to account for strength gains throughout the protocol. Participants in the exercise groups (i.e., EX+DM and EX) had 2 ± 5 days between their last exercise session and their mid- and post-assessment to minimize a carryover effect. Exercise compliance was determined by the number of sessions attended. All participants in the exercise groups (i.e., EX+DM and EX) were instructed to continue their normal physical activity outside the PRT program.

### Activity Tracking

All participants were asked to resume their usual lifestyle activity levels and were required to wear an activity monitor (ActiGraph wGT3X-BT, ActiGraph, Pensacola, FL) on their non-dominant wrist. Participants were instructed to wear the activity monitor during the course of the intervention trial from waking to bedtime and to take the monitor off only when engaged in aquatic activities. A new activity monitor was provided every 2 ± 1 weeks due to the limited (25 days) battery life. Data were uploaded to analytical software (ActiLife 6 v.6.1.3, ActiGraph, Pensacola, FL). A valid day was defined as ≥80% wear time.

### Anthropometry and Body Composition

The participants arrived at the laboratory between 7:00 a.m. and 9:00 a.m. in a fasted state [plasma osmolality = 296 ± 5.6 mOsmol/kg (Osmomat 030, Gonotec, Berlin, Germany) and total body water = 53.3 ± 6.4% (Seca 515 MBCA, Seca Group, Hamburg, Germany)]. All participants were required to avoid strenuous exercise for a 24-h period prior to all laboratory assessments. Height was assessed using a fixed stadiometer (Holtain, Crosswell, Crymych, UK). BM was measured (Seca 515 MBCA) to the nearest 0.1 kg using standardized anthropometrical procedures. Total (in kilograms) and relative (in percent) fat mass (FM) and FFM and bone mineral content (BMC) were assessed by a trained radiographer using a dual-energy X-ray absorptiometry (iDXA; Prodigy, GE Lunar, Madison, WI, with analysis software 14.10). Appendicular lean mass (ALM) was determined by adding the total arm and leg mass, and then it was adjusted for height (ALM/ht^2^).

### Submaximal Incremental Bike Test

To track changes in cardiorespiratory fitness along the experimental timeline, submaximal aerobic fitness was determined using an incremental bike test using a cycle ergometer (Corival, Lode, Groningen, Netherlands) and a metabolic cart (Vmax Encore Metabolic Cart, Carefusion, San Diego, CA). Procedures were adjusted from standard fitness testing protocols (39). The initial workload began at 1 W per kilogram of FFM (W/kg FFM) and increased by 0.5 W/kg FFM every 3 min until the participants could not maintain the speed at ≥60 rpm, they reached a rating of perceived exertion (RPE) of 15–17, and/or obtained a respiratory exchange ratio (RER) of 1.000 (40). Heart rate (HR) (Polar Electro, Kempele, Finland), RPE, *V*O_2_, and RER were measured every 3 min in real time. Cardiorespiratory fitness was expressed as the *V*O_2_ in milliliters per kilogram per minute at which the RER reached 1.000.

### Countermovement Jump

A force plate (400s+ Performance Force Plate, Fitness Technology, Adelaide, Australia) was used to measure relative muscle power (in watts per kilogram), jump height (in centimeters), and velocity (in meters per second) during a countermovement jump (CMJ) test. The participants were asked to start in a full erect standing position in the middle of the force plate and then instructed to dip to a self-selected depth and to perform a static squat jump. Hands were kept on the hips to minimize any influence of arm swing (41). The participants were asked to perform three attempts of a CMJ with a 1-min rest in between jumps. The force plate was interfaced with computer software (Ballistic Measurement System; Fitness Technology, Adelaide, Australia); the best of the three jumps was selected for further analysis.

### Gait Speed Measurement

To assess gait speed, a walking course of 4 m length was marked on the floor. The participant was instructed to walk from one end of the course to the other at their usual walking pace. The timer began as the participant started walking and the timer stopped with the first footfall after the 4-m line. The test was repeated twice and the fastest time of the two scores was recorded (1).

### Skeletal Muscle Strength Outcomes

Strength was assessed by performing a 1RM according to previously described protocols (42). During a familiarization trial, proper lifting technique was demonstrated, and then participants were familiarized with each resistance machine (Hammer Strength, LifeFitness, Sydney, Australia) by performing 8–10 repetitions of a light load (~50% of predicted 1RM). After the successful completion of a further five to six repetitions at a heavier weight selected by the instructor, the workload was increased incrementally until only one repetition with the correct technique could be completed. Participants were given 3–5 min rest in between attempts (43). The value of 1RM was the highest load that could be raised in one single repetition using the correct technique. Leg press, *lat* pull-down, and bench press exercises were measured. The 1RMs were normalized by body weight (1RM/BM). Hand grip strength (HGS) was measured using a digital hand dynamometer (Jamar® Plus+ Digital Hand Dynamometer, Sammons Preston, Bolingbrook, IL). HGS was measured in a standing position with the participants elbow by their side and flexed to 90° and a neutral wrist position. The participants were asked to apply the maximum grip strength by squeezing the dynamometer with as much force as possible using their dominant hand. This was repeated three times with a 1-min rest in between attempts. HGS was defined as the highest value for their dominant hand (44).

### Blood Collection and Analysis

Blood glucose concentration, hemoglobin, and the total and differential leukocyte counts (i.e., neutrophils, lymphocytes, and monocytes) were determined by the HemoCue system (Glucose 201+, Hb201, and WBC DIFF, respectively; HemoCue AB, Ängelholm, Sweden) in duplicate from heparin whole blood samples. The coefficients of variation (CVs) for blood glucose concentration, hemoglobin, and total leukocyte counts were 3.0, 1.5, and 4.6%, respectively. Hematocrit was determined using the capillary method in triplicate (CV = 1.1%) from heparin whole blood samples and using a microhematocrit reader (ThermoFisher Scientific). Hemoglobin and hematocrit values were used to estimate changes in plasma volume relative to baseline and used to correct plasma variables. The remaining heparin whole blood samples were centrifuged at 4,000 rpm (1,500 × g) for 10 min within 15 min of sample collection. Aliquots of heparin plasma were placed in 1.5-ml microstorage tubes and frozen at −80°C until analysis, except 2 × μl plasma was used to determine plasma osmolality in duplicate (CV = 1.1%).

Circulating concentrations of cortisol (DiaMetra, Perugia, Italy), insulin (Crux Biolab, Scoresby, Australia), IGF-1 (Crux Biolab, Scoresby, Australia), testosterone (17b-OH-4-androstene-3-one; DiaMetra, Perugia, Italy), and estradiol (17β-estradiol; DiaMetra, Perugia, Italy) were measured by enzyme-linked immunosorbent assay (ELISA). The plasma concentrations of TNF-α, IL-6, IL-8, IL-2, and IL-10 were determined by high-sensitivity multiplex ELISA (HCYTOMAG-28SK, EMD Millipore, Darmstadt, Germany). All assays were performed as per the manufacturer's specifications, with standards and controls on each plate. The CV for the analyzed circulating biomarkers was ≤ 7.2% and for the systemic inflammatory cytokine profile was ≤ 13.5%. Systemic cytokine profile was established, as previously described (45).

### Statistical Analysis

Only participants that attended ≥80% of the PRT sessions and consumed ≥80% of the DM beverage over the 12-week intervention were included in the data analysis. Based on the statistical test, mean, standard deviation, and effect size (i.e., small = 0.20, medium = 0.50, and large = 0.80) for outcomes of FFM, skeletal muscle strength, and physical performance and applying standard alpha (0.05) and beta (0.80) values, a sample size of *n* = 36 (*n* = 8 per group), using a randomized controlled design as reported in Hanach et al. (9), is estimated to provide adequate statistical power (0.80–0.99) to detect variable differences (G^*^Power 3.1, Kiel, Germany). Data in the text and tables are presented as either mean ± SD or mean and 95% confidence interval (CI), as indicated. For clarity, data in figures are presented as mean ± standard error of the mean (SEM). Only participants who completed the experimental design, adhered to the controlled intervention conditions, and with full datasets within each specific variable were included in the data analysis, as indicated in the table and figure legends. All data were checked for distribution using the Shapiro–Wilk test of normality. Variables with singular data points were examined using a one-way ANOVA or non-parametric Kruskal–Wallis test, when appropriate. Variables with multiple data points were examined using a two-way repeated-measures ANOVA with a matrix including group (DM, EX+DM, EX, and CON) and time [baseline (week 0), week 6, and week 12]. Assumptions of homogeneity and sphericity were checked, and when appropriate, adjustments to the degrees of freedom were made using the Greenhouse–Geisser correction method. Significant main effects were analyzed using a *post-hoc* Tukey's HSD test. Statistics were analyzed using SPSS statistical software (v.25.0, Chicago, IL) with significance accepted at *P* ≤ 0.05.

Furthermore, correlations between changes in the primary variables (e.g., FFM, skeletal muscle strength, power, and physical performance) and the inflammatory and hormone markers were conducted at 6 and 12 weeks. This was carried out with a Pearson's or Spearman's correlation test, based on the data distribution. Significance was accepted at *P* ≤ 0.05. Additionally, Cohen's *d* was applied to determine the magnitude of effect size for significant differences, with *d* ≥ 0.20 for small, *d* ≥ 0.50 for medium and *d* ≥ 0.80 for large effect size.

## Results

### Baseline Characteristics

The participants from this study came from a variety of sporting backgrounds, including endurance runners/race walkers (61%), cyclists (9%), aerobic gym goers (16%), or a combination of multiple activities (14%). The dropout rate for the current study was 20% between all four groups, with the dropout reasoning depicted in Figure 1. At the end of the study, the groups were composed as follows: DM, *n* = 8; EX+DM, *n* = 9; EX, *n* = 10; and CON, *n* = 10. Participants' baseline variables, based on group allocation, are summarized in Table 1. There were no significant differences in the baseline characteristic variables between groups.

**Table 1:** Baseline characteristics of the participants according to randomized group selection.

	**DM (*n* = 8)**	**EX+DM (*n* = 9)**	**EX (*n* = 10)**	**CON (*n* = 10)**	***P* value**
Males, *n*	7	6	8	7	
Females, *n*	1	3	2	3	
Age (years)	59.7 (52.9–67.0)	63.6 (57.4–70.0)	58.0 (53.0–67.0)	56.1 (51.5–60.6)	0.147
Height (m)	1.7 (1.6–1.8)	1.7 (1.6–1.7)	1.7 (1.7–1.8)	1.6 (1.6–1.7)	0.105
BM (kg)	78.3 (65.6–91.0)	71.6 (63.0–80.1)	70.0 (64.4–89.5)	67.8 (63.2–72.4)	0.333
BMI (kg/m^2^)	24.6 (22.2–27.0)	24.9 (22.0–27.8)	25.3 (22.5–28.1)	24.1 (23.0–25.3)	0.876
Self-reported structured exercise (min/week)	233 (125–340)	189 (144–264)	273 (210–336)	215 (137–293)	0.378

*Values shown are the mean (95% CI)*.

*BM, body mass; BMI, body mass index; EX+DM, exercise and high-protein milk beverage; DM, high-protein milk beverage; EX, exercise; CON, control*.

### Progressive Resistance Training and Food Provisions Compliance

In EX+DM and EX, the PRT was well-tolerated, with the average compliance for participants in the exercise program being 89% (95% CI = 85–93%), and did not differ between groups (*P* = 0.538). In DM and EX+DM, the average compliance for the high-protein dairy milk beverage provisions, according to the food diaries and milk bottle returns, was 93% (88–97%) and did not differ between groups (*P* = 0.969). Average adherence to the standardized meal plan and food provisions, based on food diaries, was 81% (76–85%) and did not differ between groups (*P* = 0.821).

### Dietary Intake

A group^*^time interaction (*P* = 0.048) was observed for energy intake, indicating a significant increase in the DM (19%) and EX+DM (22%) groups at 12 weeks compared to baseline (Table 2). Consumption of the high-protein dairy milk led to a significant increase in absolute (in grams per day; *P* = 0.001) and relative protein intake (in grams per kilogram BM per day; *P* < 0.001) in the DM and EX+DM groups at 6 weeks (38 and 35%, respectively) and 12 weeks (44 and 45%, respectively). Similarly, there was a group^*^time interaction for calcium intake (*P* = 0.007). Further analysis indicated that DM and EX+DM had a significant increase in calcium intake at 6 weeks (88 and 99%, respectively) and 12 weeks (120 and 112%, respectively) compared to baseline (Table 2). Based on the protein intake relative to BM, a group^*^time interaction effect was observed for protein (in grams per kilogram BM) at breakfast (*P* = 0.005) and dinner (*P* = 0.012) and toward significance at lunch (*P* = 0.055). Further analysis indicated that, compared to baseline, protein at breakfast significantly increased at 6 and 12 weeks in DM (≥44%) and EX+DM (≥55%) compared to EX and CON. Whereas, there was a significant decrease of relative protein intake (in grams per kilogram) at dinner in the EX+DM (50%) and EX (10%) groups at 6 and 12 weeks compared to baseline.

**Table 2:** Baseline values and the mean within-group changes at weeks 6 and 12 for total dietary energy and macronutrient intake and relative protein intake based on each meal according to randomized allocation.

	**DM (*n* = 8)**	**EX+DM (*n* = 9)**	**EX (*n* = 10)**	**CON (*n* = 10)**
**TOTAL ENERGY AND MACRONUTRIENT INTAKE**				
**Energy intake (MJ/day)**				
Baseline	8.6 (6.7–10.6)	8.1 (6.0–9.3)	9.1 (7.0–10.0)	9.6 (9.0–10.5)
6 weeks	10.0 (7.6–14.0)	9.4 (6.0–13.7)^**^	8.6 (5.7–11.4)^aa^	9.6 (4.3–13.0)
12 weeks	11.0 (8.0–14.0)^**^	9.5 (7.5–12.4)^**^^aa^	8.9 (4.3–11.8)^aa^	9.0 (6.1–12.0)^aa^
**Total protein intake (g/day)**				
Baseline	101 (81.7–120)	86.4 (67.6–105)	95.4 (77.1–113)	108 (91.0–127)
6 weeks	125 (105–148)^**^	115 (72.0–161)^**^	94.0 (54.3–119)^a^^b^	112 (52.2–159)^a^^b^
12 weeks	127 (118–152)^**^	123 (94.0–153)^**^	106 (57.0–145)^a^^b^	98 (62.0–145)^a^^b^
**Relative protein (g kg**^**−1**^ **BM day**^**−1**^**)**				
Baseline	1.3 (1.0–1.7)	1.2 (1.0–1.4)	1.4 (1.0–1.7)	1.6 (1.2–2.0)
6 weeks	1.7 (1.2–0.2.9)^**^	1.6 (1.0–2.2)^**^^aa^	1.2 (0.80–1.9)^aa^	1.6 (0.9–2.5)^aa^^cc^
12 weeks	1.8 (1.4–3.2)^**^	1.8 (1.2–1.8)^**^^aa^	1.4 (0.70–1.8)^aa^	1.4 (0.9–1.4)^aa^^cc^
**Protein (%)**				
Baseline	27.0 (19.4–34.5)	29.1 (20.6–37.5)	23.0 (17.0–27.0)	32.2 (26.8–37.6)
6 weeks	35.5 (26.0–57.0)^**^	34.0 (23.0–46.0)^**^	21.5 (10.3–31.0)^*^^a^^b^	32.3 (17.0–48.3)^a^^c^
12 weeks	37.0 (28.4–37.0)^**^	38.6 (31.0–39.0)^**^	24.4 (14.0–38.0)^*^^a^^b^	29.3 (17.5–29.3)^a^^b^^c^
**Carbohydrates (g/day)**				
Baseline	206 (128–308)	192 (80–297)	229 (108–331)	202 (155–249)
6 weeks	304 (187–305)^**^	106 (68–144)^**^	237 (187–363)^a^^b^	208 (68–305)^a^^b^
12 weeks	218 (93–330)^**^	100 (48–151)^**^	267 (171–384)^a^^b^	210 (68–348)^a^^b^
**Fat (g/day)**				
Baseline	81.6 (66.0–97.0)	70.5 (53.5–87.7)	78.6 (67.0–90.5)	92.0 (81.5–102)
6 weeks	64.0 (33.0–83.0)	16.3 (−100 to 132)	70.0 (24.0–98.0)	66.0 (44.0–122)
12 weeks	64.0 (35.0–100)	33.6 (−90.0 to 159)	75.0 (29.3–74.5)	87.4 (51.2–118)
**Calcium (mg/day)**				
Baseline	1,011 (675–1,347)	749 (394–1,104)	1,117 (807–1,426)	1,022 (789–1,256)
6 weeks	1,911 (1,467–2,458)^**^	1,629 (941–2726)^**^	1,006 (547–1613)^aa^^bb^	1,262 (582–2,074)^aa^^bb^
12 weeks	2,037 (1,449–2,849)^**^	1,695 (1,245–2,704)^**^	1,135 (605–1,592)^aa^^bb^	1,134 (462–2,151)^aa^^bb^
**RELATIVE PROTEIN INTAKE BASED ON EACH MEAL**				
**Protein at breakfast (g/kg BM)**				
Baseline	0.25 (0.10–0.40)	0.21 (0.12–0.30)	0.30 (0.15–0.40)	0.30 (0.21–0.36)
6 weeks	0.36 (0.20–0.60)^**^	0.33 (0.30–0.40)^*^	0.22 (0.0–0.50)	0.28 (0.10–0.50)
12 weeks	0.34 (0.10–0.60)^**^	0.36 (0.20–0.50)^*^	0.25 (0.10–0.40)^*^	0.31 (0.10–0.50)
**Protein at lunch (g/kg BM)**				
Baseline	0.40 (0.21–0.60)	0.31 (0.18–0.43)	0.40 (0.25–0.60)	0.37 (0.22–0.51)
6 weeks	0.50 (0.30–0.70)^*^	0.50 (0.40–0.60)^#^	0.34 (0.10–0.50)	0.45 (20–0.70)
12 weeks	0.50 (0.30–0.70)^*^	0.44 (0.30–0.50)^**^	0.31 (0.10–0.0.70)^b^	0.40 (0.10–0.60)^a^^b^^c^
**Protein at dinner (g/kg BM)**				
Baseline	0.50 (0.31–0.65)	0.64 (0.50–0.80)	0.74 (0.40–1.1)	0.64 (0.50–0.80)
6 weeks	0.40 (0.20–0.50)	0.35 (0.20–0.50)^*^	0.70 (0.30–1.0)^*^^b^	0.70 (0.20–1.2)^a^^b^
12 weeks	0.40 (0.30–0.41)	0.35 (0.20–0.50)^*^	0.60 (0.40–1.0)^b^	0.60 (0.30–1.2)^b^

*Values shown are the mean (95% CI)*.

*BM, body mass; CON, control; EX, exercise; EX+DM, exercise and high-protein dairy milk beverage; DM, high-protein dairy milk beverage*.

Within-group changes:

** P < 0.01 and

* P < 0.05 vs. baseline; Between-group changes:

aa P < 0.01 and

a P < 0.05 vs. DM;

bb P < 0.01 and

b P < 0.05 vs. EX+DM;

cc P < 0.01 and

c *P < 0.05 vs. EX*.

### Physical Activity

At baseline, the reported physical activity did not differ between groups (Table 1). The average compliance based on the wear time for the ActiGraph was 93% (90–95%) and did not differ between groups (*P* = 0.754). Based on the analysis of the accelerometer over the intervention period, there were no differences between groups for the amount of hourly kilocalories, time in sedentary, or time in light physical activity (Table 3).

**Table 3:** Average daily physical activity measured by an accelerometer over the 12-week experimental procedure.

	**DM** **(*n* = 8)**	**EX+DM** **(*n* = 9)**	**EX** **(*n* = 10)**	**CON** **(*n* = 10)**
**Sedentary time (min/day)**				
0–6 weeks	923 (844–978)	863 (672–1,035)	782 (351–933)	844 (697–941)
6–12 weeks	904 (848–978)	896 (706–1,064)	785 (152–1,017)	877 (785–985)
**Time in light physical activity (min/day)**				
0–6 weeks	281 (193–366)	340 (158–842)	368 (245–883)	280 (230–363)
6–12 weeks	282 (214–374)	352 (136–938)	359 (215–900)	275 (208–342)
**Time in moderate physical activity (min/day)**				
0–6 weeks	171 (130–218)	206 (130–315)^a^	219 (153–280)^a^	216 (174–323)^a^
6–12 weeks	170 (131–213)	210 (115–317)^a^	217 (128–310)^a^	214 (158–277)^a^
**Time in vigorous physical activity (min/day)**				
0–6 weeks	11.1 (0.0–65.2)	19.0 (0.0–58.0)	13.3 (0.0–43.8)	24.5 (0.50–55.4)
6–12 weeks	13.8 (0.0–60.0)	18.0 (0.0–47.6)	22.1 (0.0–45.3)	32.2 (0.00–57.3)
**Total steps, (*****n*****/day)**				
0–6 weeks	24,582 (9,793–11,112)	13,360 (9,723–18,505)	14,670 (11,734–17,000)	14,126 (11,787–19,928)
6–12 weeks	12,323 (10,000–15,145)	13,811 (8,742–20,175)	14,516 (10,700–17,510)	14,152 (11,220–15,686)

*Values shown are the mean (95% CI)*.

*DM, high-protein dairy milk beverage; EX, exercise; EX+DM, exercise and high-protein dairy milk beverage; CON, control*.

Between-group changes:

a *P < 0.05 vs. DM*.

### Body Composition

**Figure 3:**
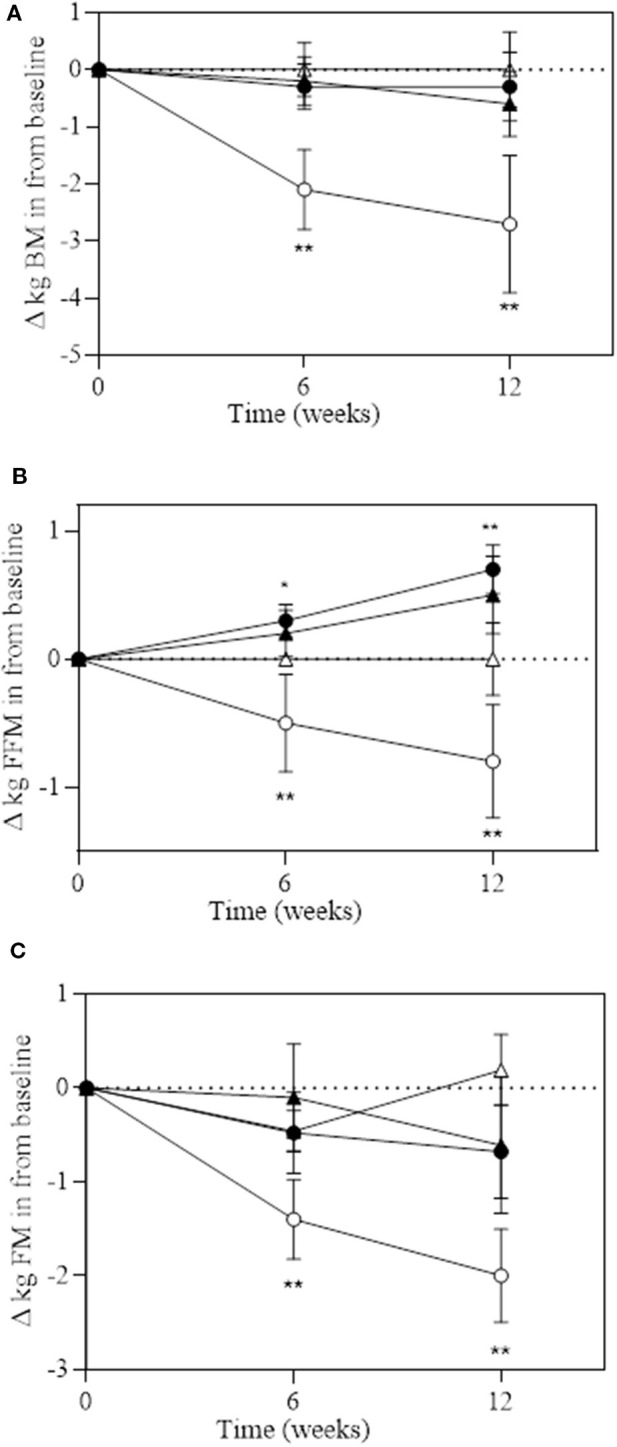
Change over 6 and 12 weeks of intervention trial from baseline for **(A)** total body mass (in kilograms); **(B)** fat-free mass (in kilograms); and **(C)** fat mass (in kilograms) according to group: DM (high-protein milk beverage, *open circle*), EX+DM (exercise + high-protein milk beverage, *filled circle*), EX (exercise, *filled triangle*), and CON (control, *open triangle*). Mean ± SEM: **P < 0.01 and *P < 0.05 vs. baseline.

A significant group^*^time interaction was observed for BM (*P* = 0.029), absolute FFM (*P* = 0.051), and absolute and relative FM (*P* = 0.013 and *P* = 0.043, respectively; Table 4 and Figure 3). BM decreased in DM at week 6 (−2.2 kg) and week 12 (−2.7 kg), which significantly reduced more than all the other groups at both time points. Absolute FFM significantly increased in EX+DM at both time points (weeks 6 and 12) and in EX at week 12. This increase was significantly greater than that in DM, which showed a significant decline in FFM at week 6 (−0.26%) and week 12 (−0.96%). Absolute FM significantly decreased over time at week 6 in DM and at 12 weeks in the DM, EX+DM, and EX groups. DM had the greatest loss in absolute FM at 6 weeks (−1.4 ± 1.2 kg) and 12 weeks (−2.1 ± 2.6 kg). At 12 weeks, there was a significant decline in relative FM of ≥1.0% in the DM, EX+DM, and EX groups compared to baseline. No significant main effects or interactions were observed for regional body composition, ALM/ht^2^, bone mineral density (BMD), or resting metabolic rate (Table 4).

**Table 4:** Baseline values and within-group changes at weeks 6 and 12 for total body and regional composition, bone mineral density, and resting metabolic rate according to randomized allocation.

	**DM (*n* = 8)**	**EX+DM (*n* = 9)**	**EX (*n* = 10)**	**CON (*n* = 10)**
**Total body FFM (%)**				
Baseline	73.0 (67.4–79.4)	69.9 (60.1–79.1)	75.7 (40.2–80.5)	76.0 (69.1–82.2)
6 weeks	75.7 (62.0–84.2)	70.1 (54.0–87.3)	75.7 (61.2–89.3)	76.5 (62.7–89.5)
12 weeks	75.5 (60.4–87.0)	70.5 (53.6–86.4)	76.6 (62.4–89.4)	76.4 (63.6–91.7)
**Total body FM (%)**				
Baseline	26.6 (20.3–32.9)	31.5 (21.6–41.4)	25.5 (19.9–31.2)	24.6 (18.1–31.1)
6 weeks	25.2 (16.0–38.0)^**^	31.5 (17.3–48.0)	25.1 (11.7–39.4)	24.4 (10.3–37.7)
12 weeks	25.0 (12.9–40.1)^**^	31.0 (14.0–47.0)^**^^a^	24.5 (11.3–37.9)^a^	24.7 (9.8–37.2)
**Arm LM (kg)**				
Baseline	5.1 (3.0–7.0)	5.1 (3.0–6.9)	6.3 (4.0–8.6)	5.6 (3.3–8.4)
6 weeks	6.0 (3.2–7.5)	5.1 (3.0–7.0)	6.3 (4.0–9.0)	5.6 (3.3–8.4)
12 weeks	6.0 (3.1–8.0)^*^	5.2 (3.0–7.2)	6.5 (3.8–9.2)	5.7 (3.3–8.4)
**Arm FM (kg)**				
Baseline	2.0 (1.6–3.7)	2.3 (1.1–4.3)	2.0 (0.8–4.0)	1.8 (1.3–2.8)
6 weeks	2.0 (1.4–3.5)	2.3 (0.8–4.1)	2.1 (0.8–3.7)	1.8 (0.7–2.8)
12 weeks	2.0 (1.1–3.4)	2.4 (1.0–4.3)	2.1 (1.0–4.0)	1.9 (0.9–3.0)
**Leg LM (kg)**				
Baseline	18.8 (12.0–23.0)	16.1 (11.0–20.4)	18.5 (14.1–24.3)	17.0 (11.8–23.2)
6 weeks	18.6 (12.2–23.0)	16.1 (11.3–21.0)	18.6 (14.2–25.0)	17.1 (12.0–24.4)
12 weeks	18.4 (12.1–22.3)	16.1 (11.0–20.5)	18.5 (14.5–24.6)	17.0 (12.0–23.1)
**Leg FM (kg)**				
Baseline	5.6 (3.6–8.0)	5.6 (3.1–10.0)	5.5 (2.4–13.0)	6.3 (2.5–14.0)
6 weeks	5.5 (4.2–7.5)	5.5 (3.0–10.1)	5.6 (2.2–13.0)	6.3 (2.3–14.0)
12 weeks	5.1 (3.4–7.3)	5.5 (3.0–10.4)	5.3 (2.0–13.0)	6.3 (2.4–14.0)
**Trunk LM (kg)**				
Baseline	26.0 (17.6–29.6)	22.6 (14.7–28.1)	26.0 (20.5–35.0)	25.1 (16.5–35.0)
6 weeks	26.0 (17.6–30.0)	23.0 (15.1–28.0)	26.0 (21.0–34.5)	25.1 (18.4–34.2)
12 weeks	26.0 (17.6–30.0)	23.0 (15.0–28.0)	26.2 (20.1–35.0)	24.2 (12.0–34.0)
**Trunk FM (kg)**				
Baseline	11.6 (4.7–21.5)	13.0 (3.5–23.6)	11.0 (2.2–20.0)	7.8 (2.7–11.0)
6 weeks	10.5 (2.3–22.0)	12.2 (2.4–23.5)	10.54 (2.0–19.6)	7.7 (2.7–11.0)
12 weeks	10.2 (3.0–21.5)	12.0 (2.2–23.0)	10.6 (1.9–20.6)	7.8 (2.2–11.0)
**ALM/ht**^**2**^				
Baseline	7.7 (6.5–9.0)	7.3 (5.1–9.1)	8.3 (5.8–11.2)	7.8 (5.6–9.4)
6 weeks	7.7 (6.6–8.6)	7.4 (5.2–9.0)	8.3 (5.8–11.2)	7.8 (5.7–9.8)
12 weeks	7.6 (6.5–8.4)	7.4 (5.2–9.0)	8.3 (5.8–11.0)	7.8 (5.7–9.3)
**BMD (g/cm)**				
Baseline	1.2 (1.0–1.4)	1.1 (0.9–1.4)	1.3 (1.0–1.6)	1.2 (0.9–1.4)
6 weeks	1.2 (1.0–1.4)	1.1 (0.9–1.4)	1.3 (1.0–1.6)	1.2 (0.9–1.4)
12 weeks	1.2 (1.0–1.4)	1.2 (0.9–1.5)	1.3 (1.0–1.4)	1.1 (0.1–1.4)
**RMR (MJ/day)**				
Baseline	6.1 (4.2–7.6)	5.5 (4.5–7.3)	6.3 (4.3–8.4)	5.7 (4.7–7.4)
6 weeks	6.1 (4.2–7.6)	5.5 (4.2–6.7)	6.3 (4.3–8.0)	5.8 (4.7–7.4)
12 weeks	6.0 (4.0–7.8)	5.4 (4.3–6.7)	6.2 (4.3–8.0)	5.6 (4.4–7.2)

*Values shown are the mean (95% CI)*.

*DM, high-protein dairy milk beverage; EX, exercise; EX+DM, exercise and high-protein dairy milk beverage; CON, control; ALM/HT, appendicular muscle mass/height; BM, body mass; BMD, bone mineral density; FFM, fat-free mass; FM, fat mass; LM, lean mass; RMR, resting metabolic rate*.

Within-group changes:

** P < 0.01 and

* P < 0.05 vs. baseline. Between-group changes:

a *P < 0.05 vs. DM*.

### Skeletal Muscle Strength

**Figure 4:**
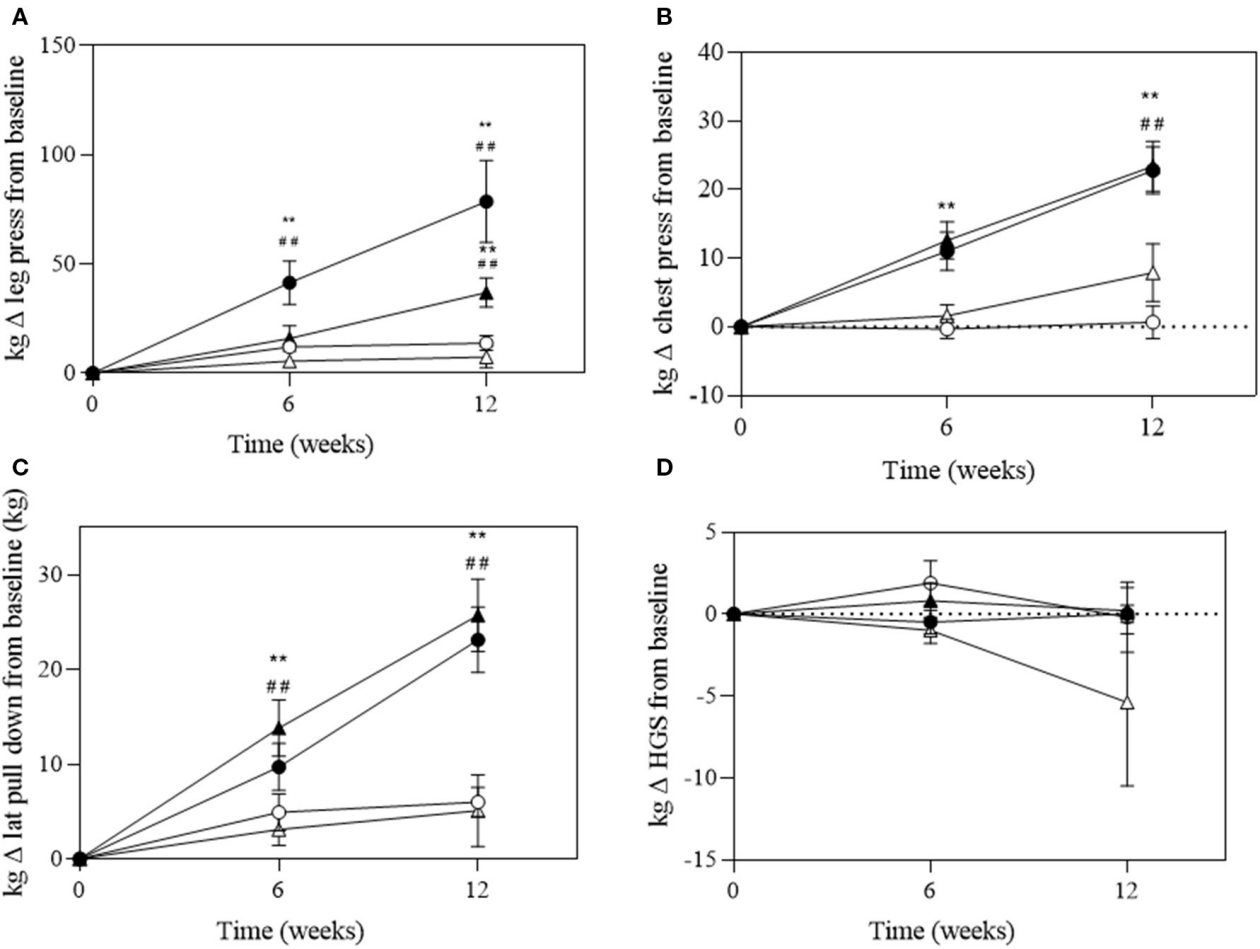
Change of absolute strength (in kilograms) at 6 and 12 weeks of intervention trial from baseline for **(A)** lower body (leg press); **(B)** upper body (chest press); **(C)** back (*lat* pull-down) strength; and **(D)** handgrip strength (HGS) according to group: DM (high-protein milk beverage, *open circle*), EX+DM (exercise + high-protein milk beverage, *filled circle*), EX (exercise, *filled triangle*), and CON (control, *open triangle*). Mean ± SEM: **P < 0.01 vs. baseline; ^##^*P* < 0.01 vs. 6 weeks.

**Figure 5:**
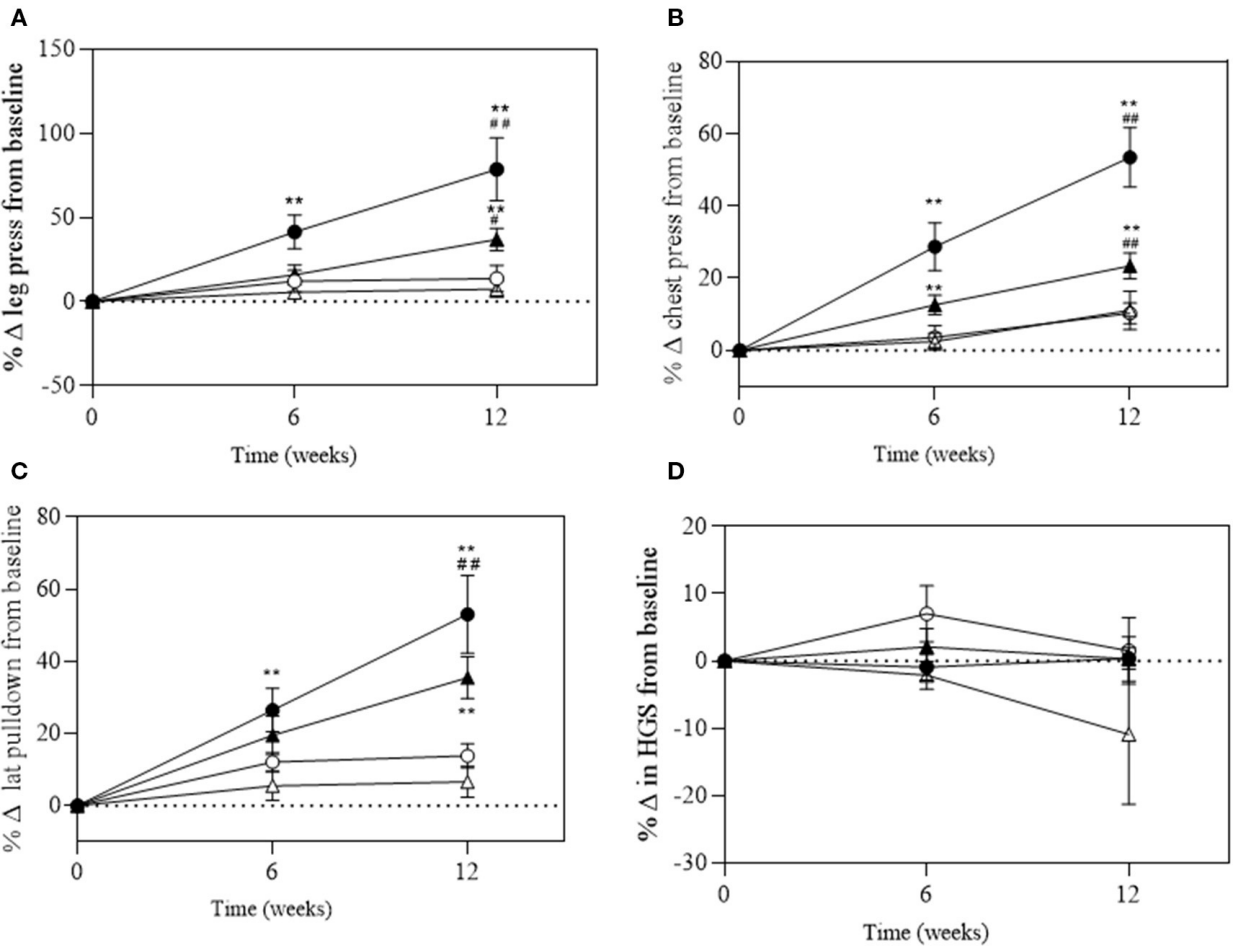
Change of relative (in kilograms per body mass, BM) at 6 and 12 weeks of intervention trial from baseline for **(A)** lower body (leg press); **(B)** upper body (chest press); **(C)** back (*lat* pull-down) strength; and **(D)** handgrip strength (HGS) according to group: DM (high-protein milk beverage, *open circle*), EX+DM (exercise + high-protein milk beverage, *filled circle*), EX (exercise, *filled triangle*), and CON (control, *open triangle*). Mean ± SEM: **P < 0.01 vs. baseline; ^##^*P* < 0.01 and ^#^*P* < 0.05 vs. 6 weeks.

There was a significant group^*^time interaction for absolute and relative maximal 1RM leg press (*P* < 0.001 and *P* = 0.006), chest press (*P* < 0.001 and *P* < 0.001), and *lat* pull-down (*P* = 0.007 and *P* < 0.001, respectively; Figure 4). A significant change in absolute maximal 1RM strength was observed in the EX+DM and EX groups at 6 and 12 weeks from baseline (Figure 4). The significant change in relative strength was observed in the EX+DM (range = 53–78%) and EX (35–36%) groups from baseline to 12 weeks (Figure 5). The change in relative 1RM strength was greater in EX+DM compared to all other groups at 12 weeks. There were no significant main effects or interactions for HGS (*P* = 0.561).

### Skeletal Muscle Power and Physical Performance

For outcomes of muscle power (i.e., CMJ), cardiorespiratory fitness (i.e., submaximal *V*O_2_), and physical performance (i.e., gait speed), there were no significant main effects or interactions from baseline to week 12 (Table 5).

**Table 5:** Baseline values and the mean within-group changes at weeks 6 and 12 in skeletal muscle power, cardiorespiratory fitness, and physical function outcomes according to randomized allocation.

	**DM (*n* = 8)**	**EX+DM (*n* = 9)**	**EX (*n* = 10)**	**CON (*n* = 10)**
**CMJ (cm)**				
Baseline	17.1 (8.4–28.0)	13.5 (3.0–21.0)	17.0 (10.3–24.2)	19.2 (10.3–26.0)
6 weeks	17.3 (7.5–22.3)	14.0 (3.3–22.1)	18.0 (12.0–24.0)	18.2 (10.3–26.0)
12 weeks	17.6 (6.7–24.0)	15.1 (2.3–29.0)	18.5 (13.3–24.0)	19.1 (11.0–25.2)
**CMJ (W/kg BM)**				
Baseline	28.9 (21.3–39.6)	25.4 (16.2–33.0)	31.7 (24.0–36.2)	29.7 (20.3–38.5)
6 weeks	28.5 (16.7–34.1)	25.4 (14.5–35.0)	31.0 (24.6–40.0)	30.2 (20.4–38.5)
12 weeks	29.0 (16.3–33.0)	28.2 (15.0–38.0)	31.4 (27.2–36.0)	30.5 (20.6–37.0)
**Gait speed (m/s)**				
Baseline	0.95 (0.80–1.20)	0.81 (0.60–1.10)	0.84 (0.70–1.00)	0.80 (0.70–0.90)
6 weeks	0.83 (0.70–1.00)	0.84 (0.70–1.10)	0.85 (0.60–1.00)	0.84 (0.70–1.00)
12 weeks	0.81 (0.60–0.90)	0.81 (0.60–0.90)	0.80 (0.60–1.00)	0.80 (0.70–0.90)
**Submaximal** ***VO**_**2**_* **(ml/kg BM/min)**				
Baseline	17.0 (11.6–22.3)	15.6 (9.7–21.5)	21.5 (17.0–26.0)	25.0 (17.0–33.0)
6 weeks	17.6 (7.0–28.3)	17.0 (5.0–28.6)	20.0 (13.0–27.0)	27.2 (15.6–39.0)
12 weeks	18.2 (8.5–27.6)	18.0 (5.7–30.0)	20.2 (12.0–28.6)	24.0 (11.0–37.1)

*Values shown are the mean (95% CI)*.

*DM, high-protein dairy milk beverage; EX, exercise; EX+DM, exercise and high-protein dairy milk beverage; CON, control; BM, body mass; CMJ, countermovement jump; FFM, fat-free mass; VO, maximal oxygen consumption*.

### Systemic Hormonal and Inflammatory Cytokine Profiles

There were no main effects or interactions observed for any of the hormonal biomarkers measured (Table 6). There was a group^*^time interaction for IL-10 (*P* = 0.016; Table 7), associated with the increase observed for the EX+DM group at 6 weeks (88%) and 12 weeks (46%). This increase was significantly higher than in all the other groups (*P* < 0.01). There were no main effects or interactions observed for any other immune biomarkers measured. There was no significant correlation between the changes in any of the primary outcomes (e.g., FFM, skeletal muscle strength, power, and physical performance) and any of the systemic hormonal and inflammatory cytokine markers.

**Table 6:** Baseline values and the mean within-group changes at weeks 6 and 12 in biochemistry and hormonal markers according to randomized allocation.

	**DM (*n* = 8)**	**EX+DM (*n* = 9)**	**EX (*n* = 10)**	**CON (*n* = 10)**
**Blood glucose (mmol/l)**				
Baseline	5.0 (4.3–5.4)	5.0 (4.5–5.5)	5.1 (5.0–5.3)	4.7 (4.1–5.4)
6 weeks	4.5 (4.0–6.0)	4.1 (3.7–6.1)	4.6 (3.2–6.2)	4.7 (3.7–5.8)
12 weeks	4.5 (3.0–6.0)	4.8 (3.2–6.5)	5.1 (4.3–6.0)	4.7 (3.4–6.1)
**Insulin (μIU/ml)**				
Baseline	8.9 (4.0–14.0)	6.4 (4.4–8.3)	5.3 (3.6–7.0)	7.4 (5.0–10.0)
6 weeks	9.2 (1.0–18.0)	6.2 (2.7–9.6)	5.2 (2.0–8.5)	6.5 (2.0–11.2)
12 weeks	5.8 (0.6–24.0)	6.6 (3.2–10.0)	5.5 (2.6–8.4)	7.6 (3.0–12.3)
**IGF-1 (pg/ml)**				
Baseline	148 (28–268)	118 (28–264)	56 (17–94)	34 (22–47)
6 weeks	171 (15–328)	220 (0–654)	115 (0–310)	57 (16–100)
12 weeks	150 (0–344)	219 (0–622)	54 (0–26)	117 (52–183)
**Estradiol (pg/ml)**				
Baseline	60.0 (18.5–138.0)	35.2 (42.0–113.0)	42.3 (7.0–91.0)	14.7 (2.0–31.4)
6 weeks	52.0 (0.0–151.0)	8.0 (0.0–145.0)	29.5 (0.0–122.0)	6.3 (0.0–34.4)
12 weeks	38.0 (0.0–147.0)	17.0 (0.0–159.0)	2.0 (0.0–128.0)	8.7 (0.0–38.0)
**Testosterone (ng/ml)**				
Baseline	2.0 (0.7–3.1)	1.3 (0.4–2.3)	2.6 (1.5–3.7)	1.4 (0.4–2.3)
6 weeks	1.9 (0.2–3.4)	1.3 (0.2–2.4)	2.7 (0.4–5.1)	1.5 (0.3–2.4)
12 weeks	2.0 (0.2–3.6)	1.2 (0.0–2.9)	2.8 (0.9–4.8)	1.5 (0.1–2.8)
**Cortisol (nmol/L)**				
Baseline	396 (241.0–551.0)	393 (227.0–559.0)	301 (229.0–372.0)	397 (179.0–615.0)
6 weeks	254 (0.0–472.0)	344 (46.2–642.0)	365 (197.0–543.0)	374 (62.0–745.0)
12 weeks	383 (68.0–698.0)	404 (135.0–674.0)	333 (214.0–451.0)	275 (0.0–67.06)

*Values are the mean (95% CI)*.

*DM, high-protein dairy milk beverage; EX, exercise; EX+DM, exercise and high-protein dairy milk beverage; CON, control; IGF-1, insulin-like growth factor-1*.

**Table 7:** Baseline values and the mean within-group changes at weeks 6 and 12 in cytokine response according to randomized allocation.

	**DM (*n* = 7)**	**EX+DM (*n* = 8)**	**EX (*n* = 8)**	**CON (*n* = 9)**
**Leukocyte** **×10**^**9**^				
Baseline	5.8 (5.4–6.4)	5.3 (4.0–7.0)	5.3 (4.6–6.0)	4.7 (4.0–5.4)
6 weeks	5.0 (3.2–7.0)	5.0 (3.0–7.5)	4.6 (3.2–6.0)	3.8 (1.5–5.0)
12 weeks	4.8 (2.3–7.4)	5.0 (3.0–7.5)	5.9 (4.0–7.8)	4.0 (1.5–6.4)
**Neutrophils** **×10**^**9**^				
Baseline	3.1 (2.3–4.0)	2.5 (1.7–3.4)	3.1 (3.0–3.5)	2.4 (2.8–3.0)
6 weeks	3.1 (1.8–4.5)	2.2 (1.0–3.5)	3.7 (2.0–3.4)	2.1 (1.5–3.6)
12 weeks	3.2 (1.4–5.2)	2.2 (0.7–3.0)	2.8 (1.5–4.5)	2.0 (1.5–3.3)
**Lymphocytes** **×10**^**9**^				
Baseline	2.5 (2.0–3.0)	2.3 (1.4–3.1)	1.9 (1.5–2.4)	1.8 (1.5–2.1)
6 weeks	2.1 (1.0–3.2)	2.1 (0.7–3.3)	1.7 (1.0–2.5)	1.3 (0.1–2.4)
12 weeks	1.7 (0.3–3.2)	2.0 (0.5–3.2)	1.7 (0.4–3.2)	1.4 (0.5–2.4)
**Monocyte** **×10**				
Baseline	0.4 (0.3–0.4)	0.3 (0.2–0.0)	0.3 (0.2–0.4)	0.4 (0.2–0.6)
6 weeks	0.4 (0.2–0.6)	0.3 (0.1–0.5)	0.3 (0.1–0.5)	0.3 (0.0–0.8)
12 weeks	0.4 (0.3–0.6)	0.3 (0.1–0.5)	0.5 (0.1–0.8)	0.3 (0.0–0.7)
**Neutrophil/lymphocyte ratio**				
Baseline	1.4 (0.7–2.0)	1.1 (0.7–1.5)	1.7 (1.4–2.0)	1.2 (0.9–1.6)
6 weeks	1.4 (0.5–2.3)	1.1 (0.5–1.8)	1.4 (0.0–2.1)	1.3 (0.6–2.1)
12 weeks	1.1 (0.5–3.0)	1.0 (0.5–1.6)	1.9 (0.9–2.9)	1.1 (0.6–1.7)
**IL-2 (pg/ml)**				
Baseline	4.2 (1.9–6.5)	3.5 (1.9–5.1)	4.2 (3.0–5.4)	5.0 (2.5–7.6)
6 weeks	3.5 (0.0–7.3)	4.0 (1.2–6.7)	4.5 (2.2–7.0)	10.7 (0.0–21.4)
12 weeks	3.4 (0.2–6.7)	3.1 (1.0–6.7)	3.6 (1.1–6.2)	6.0 (0.5–12.0)
**IL-6 (pg/ml)**				
Baseline	13.0 (0.5–25.6)	2.0 (1.1–3.0)	8.0 (2.2–13.2)	4.0 (0.3–7.2)
6 weeks	11.6 (0.0–30.6)	2.4 (0.0–4.0)	7.1 (2.2–15.2)	3.8 (0.2–8.4)
12 weeks	11.5 (0.0–21.5)	2.0 (0.6–3.7)	7.2 (0.0–16.0)	3.7 (0.0–8.1)
**IL-8 (pg/ml)**				
Baseline	10.0 (2.0–18.0)	1.9 (1.3–2.6)	5.0 (2.6–7.0)	4.8 (0.74–10.3)
6 weeks	10.5 (0.0–22.5)	1.4 (1.2–3.7)	4.6 (1.5–7.2)	4.8 (0.0–11.8)
12 weeks	9.0 (0.0–22.1)	1.0 (0.8–3.2)	4.0 (0.0–9.0)	4.4 (0.0–11.3)
**IL-10 (pg/ml)**				
Baseline	16.0 (8.2–23.5)	14.1 (2.4–25.7)	20.4 (10.0–31.1)	18.6 (6.0–31.5)
6 weeks	16.0 (8.2–23.6)^b^	23.4 (3.3–43.4)^*^	20.4 (9.8–31.0)^b^	18.7 (5.4–42.0)^b^
12 weeks	16.0 (8.0–23.5)^b^	19.0 (1.0–136.0)^*^	20.3 (9.4–31.3)^b^	18.7 (5.6–31.0)^b^
**TNF-α** **(pg/ml)**				
Baseline	1.8 (1.5–2.3)	1.8 (1.4–2.3)	2.3 (1.4–3.2)	2.4 (1.1–3.6)
6 weeks	2.0 (1.4–2.8)	2.4 (1.6–3.3)	2.1 (0.6–3.7)	2.1 (0.0–4.7)
12 weeks	2.0 (1.3–3.0)	1.8 (1.2–2.0)	3.4 (1.3–4.1)	2.0 (0.0–4.3)
**Systematic inflammatory response profile**				
Baseline	48.0 (21.0–75.0)	16.5 (3.4–29.5)	41.0 (23.5–58.1)	37.7 (14.4–61.0)
6 weeks	48.2 (6.0–90.0)	29.0 (4.4–53.5)	42.0 (12.0–72.1)	32.1 (0.7–81.4)
12 weeks	40.6 (4.2–77.0)	24.5 (0.1–49.0)	32.3 (0.0–71.0)	33.0 (0.2–72.2)

*Values are the mean (95% CI)*.

*DM, high-protein dairy milk beverage; EX, exercise; EX+DM, exercise and high-protein dairy milk beverage; CON, control; IL, interleukin; IGF-1, insulin-like growth factor-1*.

Within-group changes:

* P < 0.05 vs. baseline. Between-group changes:

a *P < 0.05 vs. DM*.

## Discussion

This study aimed to determine the independent and combined effects of a high-protein dairy milk beverage provided at breakfast and lunch (or after resistance exercise), with or without PRT, on outcomes of FFM, skeletal muscle strength and power, and physical performance in active older adults. In conflict with the hypotheses, a high-protein dairy milk beverage did not influence gains in FFM, skeletal muscle strength, power, or performance compared to the control. Whereas, in accordance with the hypotheses, a high-protein dairy milk beverage provided and consumed twice daily, in conjunction with PRT, resulted in significant increases in strength (i.e., 78% leg press, 56% chest press, and 53% *lat* pull-down) compared to PRT alone, but did not further augment changes in FFM, power, or physical performance. Moreover, the consumption of a high-protein dairy milk beverage during the PRT period resulted in significant increased levels of cytokine IL-10, suggesting an anti-inflammatory effect at this intervention, but it did not result in any anabolic hormone enhancements compared to other interventions or the control. Overall, these results suggest that the consumption of a high-protein dairy milk beverage, in combination with PRT, elicits greater effects on skeletal muscle strength outcomes than consuming the dairy milk beverage or PRT in isolation. This suggests that the DM does not seem to impact FFM, power, or physical performance any more than the CON in healthy active older adults. Therefore, high-protein dairy milk in combination with PRT may be an effective strategy in the prevention and management of age-related sarcopenia in the active aging population.

The progressive decline in strength and FMM begins to be detectable from the age of ≥50 years (1). The rate of loss that occurs in skeletal muscle mass and strength is between 1–2% and 1.5–5.0% per year, respectively (46–48). Maintaining skeletal muscle strength is a key factor to maintaining functional capacity and independent living with increasing age (1). However, even in very physical active older adults (e.g., training four to five or more sessions per week), there have been observed declines in leg strength of 3–5% per year (48). In the current study, there was a significant increase in maximal 1RM lower and upper body strength observed in both groups that received PRT (EX+DM, ≥53%; EX, ≥35%). These findings align with previous studies that show maximal 1RM leg strength increases of >25% after 12 weeks of resistance training in older adults (49, 50). The improvement in maximal relative muscle strength as measured using 1RM (e.g., leg press, chest press, and *lat* pull-down) was significantly higher in EX+DM (53–78%) compared to EX (35–36%), DM (4–7%), and CON (7–11%), indicating an interaction effect. These findings align with a recent meta-analysis which reported that protein supplementation (20 ± 18 g protein/day) further augments strength (33%), as measured by 1RM leg press, in community-dwelling older adults (≥45 years) (10). However, these findings contradict previous exercise intervention studies that have not observed protein supplementation to further increase gains in maximal 1RM leg strength during resistance exercise training in healthy community-dwelling and active older adults compared to placebo or exercise-only groups (49, 50). One possible explanation for the positive finding from the current study, compared to the aforementioned studies, is the difference in the mean age (58 ± 7 years) compared to those in the previous studies (≥70 years). These age-related discrepancies may be due to the presence of anabolic resistance and the decreased work/power capacity that occurs with increasing age (51). Secondly, the amount of daily protein consumed in the supplement groups may have been inadequate to illicit a significant strength adaptation between groups. For example, cohorts receiving additional milk servings were consuming 1.3–1.4 g kg^−1^ BM day^−1^ of protein at baseline (49, 50). Although this is higher than the recommendations for older adults (≥1.2 g kg^−1^ BM day^−1^) to treat sarcopenia, it is below (1.6 g kg^−1^ BM day^−1^) the threshold recommended to support significant changes in muscle size and strength during prolonged resistance training in healthy active adults that are novice to weight training (52–55). Furthermore, in this current study, the addition of the high-protein dairy milk beverage increased the protein intake in the EX+DM and DM groups to 1.7 and 1.9 g kg^−1^ BM day^−1^, respectively. While this is much higher than the reported amount needed for those that are novice to weight training (e.g., 1.3–1.8 g kg^−1^ BM day^−1^), the lack of a further significant effect may be influenced by the CON and EX groups that were consuming high habitual protein intakes throughout the study intervention (e.g., ≥1.4 g kg^−1^ BM day^−1^) (56). Although the EX group did habitually consume a higher amount than expected (1.4 g kg^−1^ BM day^−1^), the EX+DM group still showed a significantly greater increase in maximal strength than the EX group. This may suggest that increasing protein intake by this magnitude with PRT can lead to additional adaptations in skeletal muscle strength, as previously reported in younger adults (52). The findings of this study may indicate that higher protein (e.g., ≥1.6 g kg^−1^ BM day^−1^) intakes than those currently recommended for active older adults (≥1.2 g kg^−1^ BM day^−1^) may be required to see optimal strength adaptations. This requirement is in accordance with the nutrition guidelines for strength and power athletes for adaptations in skeletal muscle strength following resistance training (54, 55).

The current study provided participants with a 15-g protein (1.57 g leucine) dose at breakfast and lunch (or after resistance exercise). This significantly increased the relative protein intakes at those meal times (≥25%) in DM and EX+DM compared to baseline. The provision of this protein dose at these time points was based on previous reports suggesting that the distribution of protein is often inadequate at those times in older adults (56). Additionally, cross-sectional reports have indicated that, in healthy active older adults that consume sufficient protein, if one meal reaches this proposed threshold, it may be sufficient to elicit favorable results in FFM, skeletal muscle strength and power, and physical performance (57). Considering the significant increases in FFM and strength observed in EX+DM compared to other studies that only provided protein supplementation post-training (49, 50), this may suggest that the distribution of protein may be more relevant in older adults already consuming adequate amounts of total daily protein (i.e., ≥1.2 g kg^−1^ BM day^−1^). Resistance training acutely sensitizes skeletal muscle mass to anabolic effects of ingested protein (58). When considering a chronic response, PRT and nutritional supplementation have an additive effect on skeletal muscle strength (10, 57). Therefore, regular intakes of protein throughout the day increase the number of opportunities to maximally stimulate myofibrillar MPS. This accumulation of myofibrillar MPS stimulation throughout the day is likely to lead to long-term positive protein balance, which may facilitate adaptations in skeletal muscle mass and strength in active older adults. The findings of this current study may indicate that older adults who are already active and consuming adequate amounts of protein may need to consider the distribution of protein to gain further benefits from PRT.

There was a significant increase in absolute FFM in the EX+DM (1.2%) and EX (0.85%) groups at 12 weeks from baseline, whereas the DM group had a significant decrease in FFM (−1%) over the course of the intervention trial. The absence of any greater increase in FFM from the consumption of additional protein in DM aligns and conflicts with previous findings in studies that investigated PRT and protein intake in healthy community dwellers (10, 25, 27, 51) and active older adults (8, 50). The discrepancies among these studies are likely due to the large heterogeneity within the study designs, such as the use of supplementation (e.g., plain dairy milk and protein-fortified dairy milk), methods of outcomes measured (e.g., iDXA, BIA, and MRI), and participant fitness status (e.g., community-dwelling and institutionalized). Furthermore, there is emerging evidence to suggest that whole foods such as dairy may exert a greater stimulatory effect on MPS than do isolated protein supplements. For example, a review by Burd et al. (59) compared between studies the MPS response to different protein sources and showed that skim milk was greater compared to whey protein or casein. Dairy milk also contains non-protein components that act directly as anabolic signaling molecules and can regulate nutrient activity due to the “food matrix effect” (60). However, more studies are needed to confirm this, especially in older active adults. Furthermore, while there were no significant interaction effects observed on outcomes of FFM, this present study confirms that a PRT can increase FFM by 0.8–1.2% in active older adults. Although not statistically significant, this has translational practice significance in the clinical setting, as the reported loss of skeletal muscle mass observed in older adults is 1–2% per year (45–47). This could indicate a saving of 1–2 years of skeletal muscle mass with a 12-week PRT intervention and therefore has great practical and clinical significance for potentially reducing age-related muscle loss in older adults.

Aging is associated with the redistribution of FM, characterized by an increase in the abdominal region (visceral fat) and a decrease in the appendicular (mostly subcutaneous fat) (61). This study observed the greatest loss in absolute FM in DM (−2.0 kg), followed by the EX+DM and EX groups (−0.68 and −0.61 kg, respectively). The greatest loss of FM was observed in the trunk region in both the DM and EX+DM groups (5–10%). While not statistically significant, these findings align with previous exercise and protein intervention trials (62, 63). One of the most cited plausible mechanisms proposed to contribute toward the decrease in FM is that dietary protein stimulates the release of satiety hormones, increases the thermic effect of food, and stimulates protein-induced alterations in gluconeogenesis (64). In the current study, the DM group increased their RMR by 1% at 6 weeks; although this finding did not attain statistical significance, it could explain the significant FM and BM losses observed at that time point. Additionally, the greater FM loss observed in both groups that received the DM may be due, in part, to the greater calcium intake (~983 mg/day) in EX+DM (941 ± 316 mg) and DM (1,011 ± 331 mg). Increasing dietary calcium, either through whole foods or supplementation, has been shown to increase fat oxidation (65) and fat excretion in the digestive tract (66). However, the findings of this current study contrast with the findings of Kukuljan et al. (25), who observed a significant increase in FM (1.3 kg) in healthy older community dwellers (50–70 years) who received a fortified milk beverage to consume twice daily for 18 months. The FM gain observed in the study by Kukuljan et al. (25) was likely due to the addition of extra energy intake (836 kJ, 200 kcal), which may have led to the excess energy intake leading to the gain in FM. This highlights one of the strengths of the current study, where there was a provision of food for 12 weeks, controlled for energy intake and provided 100% of the estimated total daily energy requirements, and estimated protein intakes for the participants in both the EX+DM and DM groups. Dietary control and food provisions also accounted for the extra energy provided by the high-protein dairy milk beverage. It is important to acknowledge that other studies have failed to provide adequate dietary controls. Therefore, the increase in calcium and protein intakes from DM may potentially explain the increased loss in FM observed in this current study despite the participants' balanced energy intake. Lastly, in relation to physical activity, DM had the lowest moderate physical activity and the highest sedentary physical activity compared to the other groups (Table 3). Therefore, the losses of FM and BM observed in DM cannot be due to the differences in habitual physical activity leading to increases in energy expenditure. Overall, there were no significant differences for markers (e.g., RMR, calcium, physical activity, and protein intake) individually, but combined they could have a substantial effect, which may explain the significant decreases in BM and FM observed in DM compared to the other groups.

The finding that the high-protein dairy milk beverage did not enhance the effects of PRT measures of HGS and performance outcomes (e.g., gait speed, countermovement jump, and cardiorespiratory fitness) is consistent with two meta-analyses that have reported mixed findings with regard to the benefit of additional dairy milk protein or protein supplementation on outcomes of physical performance (9, 67). In the current study, the lack of a significant change is likely due to the examination of active older adults who have physical performance measures higher than community dwellers or frail older adults, who are the typical participants in sarcopenia research (68). This highlights the lack of research in active older adults who are still prone to age-related sarcopenia (68). For example, Dulac et al. (69) recruited sedentary (<120 min/week of physical activity) older (69 ± 7 years) males and found significant gains in all groups for HGS (4–10%) and gait speed (−4 to −5%). Similarly, Daly et al. (63) found a significant increase in HGS (10–14%) and gait speed (−3%) in inactive (<7,500 steps/day) females. Considering that the self-reported baseline physical activity level for this current study was 227 ± 31 min/week, which is higher than the level of physical activity that is considered “active” for older adults [e.g., 150 min/week of light- to moderate-intensity or 75 min/week of vigorous-intensity physical activity; (11)] and higher than the previously mentioned studies, could indicate that the active older adults in this current study reached a “ceiling effect” in the outcomes of performance, similarly observed in high-functioning and highly trained older adults (68). For example, the average HGS values at baseline were 37 and 42 kg, for females and males, respectively. These are higher than the previous findings in community-dwelling females (≥27 kg) (69) and males (≥38 kg) (70). Moreover, gait speed has been found to have a non-linear relationship between leg strength, as indicated by a wide population variance [e.g., 22%; (68)]. Therefore, any changes in skeletal muscle mass and strength in active older adults are unlikely to show an improvement in gait speed or HGS. A review by Beaudart et al. (70) proposed that a gait speed test over a course of 400 m would be more clinically relevant and sensitive to detect changes in active older adults than would a 4-m distance. Previous works have suggested that measures of muscle power (e.g., CMJ) should be considered more clinically relevant in the active older population due to power declining at a faster rate than strength (51). Within this current study, there was an average increase by 10% in jump height in the groups that received PRT. Although this finding was not statistically significant, it aligns with Daly et al. (63), where a significant change in the CMJ height with a change of 3–6% was reported. The difference of results is likely due to the much larger sample size per group (*n* = 108) within the study by Daly et al. (63). Overall, the measurements of HGS and gait speed have been used as valid measurements in the clinical setting to detect age-related declines related to sarcopenia and may be more useful for use as an initial screening of participants, but may not be sensitive enough to detect meaningful changes in an intervention trial in an older population that is physically active.

Considering the role of systemic inflammatory responses in the pathophysiology of age-related sarcopenia (31, 32), the current study employed a human cyto/chemokine panel to determine intervention-induced changes in these immune response markers. Using a high-sensitivity multiplex assay, the results of the current study found a significant increase in the plasma IL-10 concentration in the EX+DM group (81%) at 12 weeks, which was greater than those in the EX (−6%), DM (−5%), and CON (−8%) groups and without any significant changes to any of the other cytokine markers measured (e.g., TNF-a, IL-2, IL-6, and IL-8). Previous studies have indicated that the anti-inflammatory cytokine IL-10 is the most sensitive cytokine marker in response to exercise stress, unlike the pro-inflammatory (TNF-a) and response (IL-6 and IL-8) cytokines which showed no to minimal responses to acute exercise (71–77). For example, studies that evaluate the cytokine response to resistance training in older adults have reported a significant increase in the resting plasma IL-10 concentration (23–50%) following 16–24 weeks of resistance training compared to the controls (no training) (35, 36). The significant difference in the increase of plasma IL-10 concentration, suggesting a greater systemic anti-inflammatory effect in EX+DM compared to the EX group, is a novel finding, and the mechanism/s for such an outcome are yet unknown. Some plausible mechanisms have largely been explored in *in vitro* and *in vivo* models and could be possibly explained by the addition of the high-protein milk beverage. In particular, the addition of branched-chained amino acids (BCAAs) found in dairy milk acts as a substrate for the synthesis of short-chained fatty acids (SCFAs) such as butyrate (78). Butyrate and other derived SCFAs from BCAAs (e.g., isobutyric acid, 2-methylbutyric acid, and isovaleric acid) increase the expressions of IL-10 lymphocyte cells in the gut (79). Additionally, other constituents of dairy milk have immunomodulatory and anti-inflammatory properties (e.g., immunoglobulins, lactoferrin, and α-lactalbumin), which may act upon cytokine upregulation or downregulation (23). Galactooligosaccharides (GOS), which are prebiotic substrates derived from lactose found in dairy milk (80–82), have been found to promote the increase in the bacterial counts of bifidobacteria and lactobacilli, which have anti-inflammatory effects (81). GOS derived from dairy milk were found to play a direct role in the regulation of CD4^+^ T cells, which are involved in the proliferation of IL-10 cytokines; however, this study was limited in animal models (82). Lastly, research has previously found that individuals who consume diets that are higher in GOS following a bout of strenuous physical activity showed lower levels of intestinal fatty acid binding protein (I-FABP; an indirect marker of intestinal epithelial injury and the regulatory point for luminal bacterial endotoxin translocation and subsequent systemic inflammatory responses) compared to those that followed a low-GOS diet [e.g., low FODMAPs; (71)]. These studies, along with our current findings, indicate a potential link between protein intake and skeletal muscle health in older adults. However, further research is needed to understand the mechanistic potential for dairy milk.

Anabolic resistance in aging individuals may be due to the changes in systemic anabolic hormones with increasing age (e.g., testosterone and IGF-1), which has a direct correlation with the onset of sarcopenia (83, 84). In addition, the decrease in estrogen levels associated with menopause may also play a role in the decline in skeletal muscle mass and skeletal muscle strength in aging females (85). In the current study, there were no significant changes in the outcomes related to systematic resting hormonal markers in any of the groups. Previous studies have found that resistance training alone can increase the circulating levels of IGF-1 (86) and testosterone (87) in older adults. In contrast, other studies have not found such effect (88, 89). In the current study, there was an increase in testosterone in both groups that received PRT (>7%). However, there was only an increase in IGF-1 in the EX+DM group (80%) at 12 weeks from baseline. West and Phillips (90) found in young males (18–30 years) that the associated effect (e.g., increase in skeletal muscle strength) of resistance training on circulating anabolic hormones was modest and explained 8–12% of the variance for changes in lean skeletal muscle. Considering that older adults have lower resting anabolic circulating hormones than their younger counterparts, the effect of PRT may be even less. Nonetheless, in older adults, even a modest effect on skeletal muscle strength or skeletal muscle may have practical implications as even small improvements could result in increased functional capacity for those at risk of sarcopenia.

Overall, the strengths of this study lie in its randomized controlled design, high study retention (≥80%) and compliance rate (≥80%) to the intervention, and in the comprehensive outcomes measured, including FFM, strength, power, and physical performance, accounting for outcomes that may be more relevant in an active aging cohort (e.g., CMJ). This study also controlled for variables such as dietary intake through using food provisions and monitoring food intake through diaries. Physical activity was monitored over the course of the clinical trial to ensure that changes in outcomes were due to the exercise intervention and not due to the participants increasing habitual exercise outside the trial, which other studies have failed to implement. The measurement of blood markers (e.g., cytokines and hormones) provided an extensive insight into the pathophysiology and potential mechanisms of the interventions. Previous nutrition and exercise intervention studies have shown significant interaction effects on sarcopenia outcomes (i.e., FFM, strength, and performance) with a study size of 6–196 participants per group, in two to four group studies (8, 9). In the present study, a larger sample size may have accounted for identifying subtle significant differences between the outcomes measured. From a clinical and practical perspective, these smaller changes that may have been observed in a larger sample size would possibly be of no clinical relevance beyond the magnitude of change that has already been observed in this current study that was sufficiently statistically powered. One limitation that should be acknowledged is that physical activity at baseline was self-reported. Therefore, it is unknown whether non-exercise activity or exercise activity, which can be significant components of energy expenditure, increased during the intervention period. This could have resulted in an increased energy deficit leading to more significant weight loss, as observed in DM (91). However, the most important limitation of this study is the large habitual protein intakes in the EX and CON groups (e.g., 1.4–1.6 g kg^−1^ BM day^−1^). These participants were not provided a control diet based on previous studies that have suggested that up to 50% of active older adults do not meet the protein requirement of 1.2 g kg^−1^ BM day^−1^ (92). Therefore, an assumption was made that protein intake would be lower and unevenly distributed than our dietary intervention. However, the protein intake between groups was comprehensively managed, assessed, and analyzed, and a strength in comparison to previously published investigations of a similar nature (8, 9). Furthermore, unlike previous studies that used isolated protein supplementation, this study used whole foods (i.e., dairy milk), which are commercially available and accessible. The addition of 500 ml of dairy milk translates to two additional servings of dairy, consistent with the Australian Guidelines to Healthy Eating (93), which may provide a cost-effective solution to providing a high-quality protein source and subsequent amino acids and were well-tolerated (compliance, 93 ± 8%).

## Conclusion

This study showed that a daily consumption of two high-protein milk beverages at breakfast and lunch (or after PRT) significantly enhanced the effects of PRT on skeletal muscle strength outcomes in active older adults who already have high levels of protein intake (≥1.2 g kg^−1^ BM day^−1^). There was a significant increase in FFM following the PRT, but no augmented effect with the high-protein dairy milk beverage. There was a significant decrease in FM in those consuming the high-protein milk beverage. Potentially, the influence from the protein or other constituents of the high-protein milk beverage (e.g., calcium) may have contributed to the significant reduction in FM observed. Additionally, EX+DM led to a significant increase in resting anti-inflammatory cytokine (i.e., IL-10), which all may play a significant role in improving skeletal muscle mass and strength outcomes in active older adults (e.g., inflammaging). Finally, these results suggest that consuming a high-protein milk beverage at times that are usually inadequate in protein with PRT can facilitate gains in muscle strength, FFM, and reductions in FM and is well-tolerated by active older adults. Overall, the findings of this study are novel and define opportunities for future interventional studies examining age-related sarcopenia in the healthy active aging population.

## Data Availability Statement

The original contributions presented in the study are included in the article/supplementary material, further inquiries can be directed to the corresponding author/s.

## Ethics Statement

The studies involving human participants were reviewed and approved by Monash University Human Research Ethics Committee. The patients/participants provided their written informed consent to participate in this study.

## Author Contributions

ZH and RC contributed toward the original research idea. ZH, RC, and JP contributed toward development of the experimental design. ZH, RC, and AP contributed toward various aspects of data collection, sample collection, and analysis. ZH and RC contributed toward the analysis of the raw data. ZH was responsible for the initial manuscript draft. All authors contributed to the critical review of the manuscript and approved the final manuscript for submission.

## Conflict of Interest

The authors declare that the research was conducted in the absence of any commercial or financial relationships that could be construed as a potential conflict of interest.

## Abbreviations

BIA: bioelectrical impedance
BMI: body mass index
BM: body mass
CI: confidence interval
CON: control
DEXA: dual-energy X-ray absorptiometry
EAA: essential amino acid
EX: exercise
FM: fat mass
FFM: fat-free mass
DM: high-protein dairy milk beverage
MPS: muscle protein synthesis
MRI: magnetic resonance imaging
PRT: progressive resistance training
SD: standard deviation.
